# Supplementary material for: Work-related stressors and mental health among LGBTQ workers: Results from a cross-sectional survey
Source: PLoS One. 2022 Oct 25;17(10):e0275771. doi: 10.1371/journal.pone.0275771 (PMC9595555; doi:10.1371/journal.pone.0275771)
Supplement: S1 File — (DOCX) [file pone.0275771.s001.docx]

Supporting Information

1. Full results of univariate ordered logistic regression models

**Mental health and gender identity**

Number of observations = 531

LR chi2(2) = 39.55

Prob > chi2 = 0.0000

Pseudo R2 = 0.0361

Log likelihood = -528.75386

|  | Odds ratio | Std. err. | Z | P > [z] | [95% conf. interval] | |
| --- | --- | --- | --- | --- | --- | --- |
| **Gender identity** |  |  |  |  |  |  |
| Cisgender woman | 2.145556 | 0.4152183 | 3.94 | 0.000 | 1.468288 | 3.135223 |
| Trans | 4.287955 | 1.026093 | 6.08 | 0.000 | 2.68263 | 6.853932 |

**Mental health and age range**

Number of observations = 531

LR chi2(2) = 45.74

Prob > chi2 = 0.0000

Pseudo R2 = 0.0417

Log likelihood = -525.66012

|  | Odds ratio | Std. err. | Z | P > [z] | [95% conf. interval] | |
| --- | --- | --- | --- | --- | --- | --- |
| **Age range** |  |  |  |  |  |  |
| Under 35 | 3.58201 | 0.7054025 | 6.48 | 0.000 | 2.435008 | 5.269304 |

**Mental health and industry**

Number of observations = 531

LR chi2(2) = 26.80

Prob > chi2 = 0.0000

Pseudo R2 = 0.0244

Log likelihood = -535.12984

|  | Odds ratio | Std. err. | Z | P > [z] | [95% conf. interval] | |
| --- | --- | --- | --- | --- | --- | --- |
| **Industry** |  |  |  |  |  |  |
| White collar public/private | 1.363115 | 0.3783986 | 1.12 | 0.264 | 0.7911158 | 2.348684 |
| Low-wage services | 3.140819 | 0.9191752 | 3.91 | 0.000 | 1.769844 | 5.573795 |

**Mental health and income**

Number of observations = 531

LR chi2(2) = 81.36

Prob > chi2 = 0.0000

Pseudo R2 = 0.0742

Log likelihood = -507.85265

|  | Odds ratio | Std. err. | Z | P > [z] | [95% conf. interval] | |
| --- | --- | --- | --- | --- | --- | --- |
| **Income** |  |  |  |  |  |  |
| Under $20,000 | 5.054945 | 0.9361014 | 8.75 | 0.000 | 3.516304 | 7.266854 |

**Mental health and employment relationship**

Number of observations = 531

LR chi2(2) = 34.61

Prob > chi2 = 0.0000

Pseudo R2 = 0.0315

Log likelihood = -531.22487

|  | Odds ratio | Std. err. | Z | P > [z] | [95% conf. interval] | |
| --- | --- | --- | --- | --- | --- | --- |
| **Employment relationship** |  |  |  |  |  |  |
| Non-standard | 2.001035 | 0.3366706 | 4.12 | 0.000 | 1.438936 | 2.782709 |
| Unemployed | 25.22141 | 20.31542 | 4.01 | 0.000 | 5.201646 | 122.2919 |

**Mental health and workplace environment**

Number of observations = 531

LR chi2(2) = 38.98

Prob > chi2 = 0.0000

Pseudo R2 = 0.0355

Log likelihood = -529.03827

|  | Odds ratio | Std. err. | Z | P > [z] | [95% conf. interval] | |
| --- | --- | --- | --- | --- | --- | --- |
| **Workplace environment** |  |  |  |  |  |  |
| Negative/unsure | 3.155228 | 0.5866303 | 6.18 | 0.000 | 2.191657 | 4.542437 |

**Mental health and social life affected by work**

Number of observations = 531

LR chi2(2) = 27.80

Prob > chi2 = 0.0000

Pseudo R2 = 0.0253

Log likelihood = -534.62904

|  | Odds ratio | Std. err. | Z | P > [z] | [95% conf. interval] | |
| --- | --- | --- | --- | --- | --- | --- |
| **Social life affected by work** |  |  |  |  |  |  |
| Often/always | 2.740012 | 0.5265701 | 5.24 | 0.000 | 1.880054 | 3.993326 |

**Mental health and substance use to cope with work**

Number of observations = 531

LR chi2(2) = 7.29

Prob > chi2 = 0.0069

Pseudo R2 = 0.0066

Log likelihood = -544.8868

|  | Odds ratio | Std. err. | Z | P > [z] | [95% conf. interval] | |
| --- | --- | --- | --- | --- | --- | --- |
| **Substance use to cope with work** |  |  |  |  |  |  |
| Uses substances to cope with work | 1.559628 | 0.2577385 | 2.69 | 0.007 | 1.128118 | 2.156192 |

**Mental health and alcohol use**

Number of observations = 531

LR chi2(2) = 2.39

Prob > chi2 = 0.1222

Pseudo R2 = 0.0022

Log likelihood = -547.3361

|  | Odds ratio | Std. err. | Z | P > [z] | [95% conf. interval] | |
| --- | --- | --- | --- | --- | --- | --- |
| **Alcohol** |  |  |  |  |  |  |
| Yes | 1.340157 | 0.2533407 | 1.55 | 0.121 | 0.925223 | 1.941178 |

**Mental health and race/ethnicity**

Number of observations = 529

LR chi2(2) = 3.78

Prob > chi2 = 0.2858

Pseudo R2 = 0.0035

Log likelihood = -543.30315

|  | Odds ratio | Std. err. | Z | P > [z] | [95% conf. interval] | |
| --- | --- | --- | --- | --- | --- | --- |
| **Race/ethnicity** |  |  |  |  |  |  |
| Black | 0.7646105 | 0.2665608 | -0.77 | 0.441 | 0.3860929 | 1.514219 |
| Indigenous | 1.191686 | 0.2894567 | 0.72 | 0.470 | 0.7402998 | 1.918298 |
| Other racialized | 0.5103119 | 0.2257724 | -1.52 | 0.128 | 0.2144108 | 1.214576 |

2. Full results of multivariate ordered logistic regression model

Number of observations = 531

LR chi2(2) = 176.24

Prob > chi2 = 0.0000

Pseudo R2 = 0.1606

Log likelihood = -460.41274

|  | Odds ratio | Std. err. | Z | P > [z] | [95% conf. interval] | |
| --- | --- | --- | --- | --- | --- | --- |
| **Gender identity** |  |  |  |  |  |  |
| Cisgender women | 1.914135 | 0.4026965 | 3.09 | 0.002 | 1.26735 | 2.891004 |
| Trans | 3.007043 | 0.7722983 | 4.29 | 0.000 | 1.817715 | 4.974547 |
| **Age range** |  |  |  |  |  |  |
| Under 35 | 2.078564 | 0.4782761 | 3.18 | 0.001 | 1.324045 | 3.263054 |
| **Industry** |  |  |  |  |  |  |
| White collar public/private | 1.667849 | 0.5051249 | 1.69 | 0.091 | 0.9212169 | 3.019616 |
| Low-wage services | 1.981116 | 0.6380835 | 2.12 | 0.034 | 1.053786 | 3.724494 |
| **Income** |  |  |  |  |  |  |
| Under $20,000/year | 2.851526 | 0.6218539 | 4.80 | 0.000 | 1.859728 | 4.372253 |
| **Social life affected by work** |  |  |  |  |  |  |
| Often/always | 1.995949 | 0.416386 | 3.31 | 0.001 | 1.326098 | 3.004162 |
| **Employment relationship** |  |  |  |  |  |  |
| Non-standard | 1.071519 | 0.2067776 | 0.36 | 0.720 | 0.734071 | 1.564088 |
| Unemployed | 9.453627 | 8.460568 | 2.51 | 0.012 | 1.636107 | 54.62423 |
| **Workplace environment** |  |  |  |  |  |  |
| Negative/unsure | 2.245742 | 0.4510187 | 4.03 | 0.000 | 1.514993 | 3.328964 |
| **Substance use to cope with work** |  |  |  |  |  |  |
| Uses substances to cope | 1.479888 | 0.2696069 | 2.15 | 0.031 | 1.035515 | 2.114956 |
